# Deep learning‐based cone‐beam CT motion compensation with single‐view temporal resolution

**DOI:** 10.1002/mp.17911

**Published:** 2025-06-04

**Authors:** Joscha Maier, Stefan Sawall, Marcel Arheit, Pascal Paysan, Marc Kachelrieß

**Affiliations:** ^1^ Division of X‐Ray Imaging and Computed Tomography German Cancer Research Center (DKFZ) Heidelberg Germany; ^2^ Medical Faculty Heidelberg Heidelberg University Heidelberg Germany; ^3^ Varian Medical Systems Imaging Laboratory, GmbH Baden‐Daettwil Switzerland

**Keywords:** 4D CBCT, deep learning, motion compensation

## Abstract

**Background:**

Cone‐beam CT (CBCT) scans that are affected by motion often require motion compensation to reduce artifacts or to reconstruct 4D (3D+time) representations of the patient. To do so, most existing strategies rely on some sort of gating strategy that sorts the acquired projections into motion bins. Subsequently, these bins can be reconstructed individually before further post‐processing may be applied to improve image quality. While this concept is useful for periodic motion patterns, it fails in case of non‐periodic motion as observed, for example, in irregularly breathing patients.

**Purpose:**

To address this issue and to increase temporal resolution, we propose the deep single angle‐based motion compensation (SAMoCo).

**Methods:**

To avoid gating, and therefore its downsides, the deep SAMoCo trains a U‐net‐like network to predict displacement vector fields (DVFs) representing the motion that occurred between any two given time points of the scan. To do so, 4D clinical CT scans are used to simulate 4D CBCT scans as well as the corresponding ground truth DVFs that map between the different motion states of the scan. The network is then trained to predict these DVFs as a function of the respective projection views and an initial 3D reconstruction. Once the network is trained, an arbitrary motion state corresponding to a certain projection view of the scan can be recovered by estimating DVFs from any other state or view and by considering them during reconstruction.

**Results:**

Applied to 4D CBCT simulations of breathing patients, the deep SAMoCo provides high‐quality reconstructions for periodic and non‐periodic motion. Here, the deviations with respect to the ground truth are less than 27 HU on average, while respiratory motion, or the diaphragm position, can be resolved with an accuracy of about 0.75 mm. Similar results were obtained for real measurements where a high correlation with external motion monitoring signals could be observed, even in patients with highly irregular respiration.

**Conclusions:**

The ability to estimate DVFs as a function of two arbitrary projection views and an initial 3D reconstruction makes deep SAMoCo applicable to arbitrary motion patterns with single‐view temporal resolution. Therefore, the deep SAMoCo is particularly useful for cases with unsteady breathing, compensation of residual motion during a breath‐hold scan, or scans with fast gantry rotation times in which the data acquisition only covers a very limited number of breathing cycles. Furthermore, not requiring gating signals may simplify the clinical workflow and reduces the time needed for patient preparation.

## 1 INTRODUCTION

In recent years, cone‐beam computed tomography (CBCT) has found wide application in various fields of medical imaging, including dentistry,^1^ orthopedics,^2^ interventional radiology,^3^ and image‐guided radiotherapy.^4^ Among the main reasons for this trend is the high flexibility of CBCT, its high spatial resolution, comparably low cost, and constantly improving image quality. However, on the downside, CBCT's poor temporal resolution poses a major challenge for any application dealing with patient motion. Since the gantry speed is often as low as 60 s per rotation, the acquisition time is large compared to the time scale of typical motion patterns. As a result, anatomical regions affected by motion appear blurred or distorted in the corresponding CBCT reconstructions. Therefore, several approaches have been proposed to address this issue.

Considering non‐periodic motion such as involuntary muscle motion, twitching, or swallowing, existing approaches are designed to provide a single artifact‐free volume. This is typically achieved by performing some sort of warped backprojection that compensates for the present motion. In this context, different strategies have been proposed to derive the motion estimate according to which the backprojection matrices are modified. These strategies include the use of fiducial markers,^5^ the use of projection domain consistency conditions,^6^ 2D/3D registration to a motion‐free prior volume,^7^, ^8^ iterative re‐projection schemes,^9^ as well the minimization motion artifact metrics.^10^, ^11^

Applications dealing with cardiac or respiratory motion, on the other hand, rather rely on 4D (3D + time) reconstructions, that is, temporal sequences of 3D reconstructions representing the patient in consecutive states of the motion cycle. Here, these reconstructions typically rely on some sort of retrospective gating strategy. For that purpose, a motion surrogate signal, such as the displacement of a breathing belt (respiratory motion) or an ECG (cardiac motion), is acquired along with the CBCT scan. In that way, any x‐ray projection of the CBCT scan can be assigned to a certain motion phase. Sorting the projections according to their phase into different motion bins (referred to as gating) and reconstructing them separately yields the desired time‐resolved representation of the motion cycle.^12^, ^13^ However, as each bin only uses a subset of the acquired projections, the gated reconstructions may show strong angular undersampling artifacts. One option to address this issue is to use hardware‐based approaches which adapt the gantry rotation speed and the acquisition of projection images to the patient's respiration.^14^, ^15^, ^16^

Other software‐based approaches rather make use of 4D reconstruction algorithms or rely on motion compensation strategies. The former are usually based on iterative reconstruction schemes that incorporate some sort of spatio‐temporal regularization,^17^, ^18^, ^19^, ^20^, ^21^ make use 4D image filter operations,^22^, ^23^ or employ deep learning to cope with the limited amount of data.^24^, ^25^, ^26^ Motion compensation, on the other hand, aims to reconstruct any motion phase from all available data. In particular, this is realized by estimating displacement vector fields (DVFs) that model inter‐phase motion. Using these DVFs, any phase can be deformed into an arbitrary reference phase such that the sum of the deformed phases yields the final motion‐compensated reconstruction. While early motion compensation approaches required properly sampled prior CT scans to estimate the DVFs,^27^, ^28^, ^29^ later approaches were optimized to estimate DVFs directly from the gated reconstructions using additional regularization techniques,^30^, ^31^, ^32^, ^33^, ^34^, ^35^, ^36^, ^37^ or even from an initial 3D reconstruction.^38^

However, despite recent advances, there are still several downsides of current approaches. Most of them are directly linked to the gating process which implicitly assumes a periodicity and cannot be applied to irregular motion patterns. Furthermore, gating requires additional patient preparation and leads to a loss of temporal resolution by sorting the projections into a limited number of phase bins. Gating‐free approaches, on the other hand, are currently not designed to provide 4D reconstructions but only a single artifact‐free reconstruction.

Therefore, we propose a novel single angle‐based motion compensation (SAMoCo) approach to overcome these drawbacks. The general idea of the proposed approach is inspired by the partial‐angle based motion compensation (PAMoCo), our prior work on coronary artery motion compensation in cardiac CT.^39^, ^40^ To increase temporal resolution, the PAMoCo divides the scan range into several consecutive partial angles, reconstructs them separately within a small patch around the coronary artery, deforms them to the central motion state of the scan according to a motion model, and finally sums the deformed images to obtain a motion‐compensated reconstruction. Here, we adapt this strategy to be applicable to 4D CBCT. In particular this includes the backprojection and deformation of single projection views instead of multiple views to account for CBCT's low gantry rotation speed, the estimation of dense, non‐constrained vector fields for the entire field‐of‐view instead of a single vector at the coronary artery, and the estimation of vector fields between arbitrary motion states. In that way a distinct reconstruction can be provided for any motion state that had occurred during the CBCT scan. Since motion is estimated without any further assumptions or constraints on the underlying motion, the proposed approach applies to periodic and non‐periodic motion patterns and provides single‐view temporal resolution for 3D and 4D applications.

## 2 MATERIALS AND METHODS

### 2.1 SAMoCo

#### 2.1.1 General concept

In the following a patient is described in terms of its time‐dependent distribution of the attenuation coefficient f(r,t). During a CBCT scan, x‐ray projections of f(r,t) are acquired at N successive angles $\vartheta \in \{\vartheta \left(t_{1}\right),\text{…},\vartheta \left(t_{N}\right)\}$ that correspond to the time points $t\in \{t_{1},\text{…},t_{N}\}$. Using the short form $f_{n}{\equiv f\left(r,t\right)|}_{t=t_{n}}$, the projection $p_{n}$ corresponding to the n ^th^ view (since we assume every view to have an individual motion state, n also refers to the motion state) is given as:

(1) $$p_{n}=X_{n}f_{n},$$

where X denotes the x‐ray transform operator and $X_{n}$ its n ^th^ component corresponding to the n ^th^ view of the scan. Since different motion states in the set of projections $\left\{p_{n}\right\}$ are superimposed during the CT reconstruction process, the corresponding image (i.e., $X^{-1}p$) suffers from motion artifacts. To address this issue, existing approaches typically sort projections corresponding to similar motion states into K motion bins that are reconstructed independently to derive a set of gated reconstructions:

(2) $${\tilde{f}}_{k}=\sum_{n=1}^{N}X_{n}^{-1}{\mathrm{II}}_{kn}p_{n}.$$

Here $X_{n}^{-1}$ is the n ^th^ component of reconstruction operator and ${\mathrm{II}}_{kn}$ is a gating function that equals one if the motion states $f_{k}$ and $f_{n}$ fulfill a certain similarity criterion and zero otherwise.

Current motion compensation approaches operate on these gated reconstructions by estimating motion in terms of a deformable transformation ${\tilde{T}}_{l}^{k}:r\to r+{\tilde{u}}_{l}^{k}\left(r\right)$, which consists of the identity mapping and a DVF ${\tilde{u}}_{l}^{k}$ such that:

(3) $${\tilde{f}}_{k}={\tilde{T}}_{l}^{k}\circ {\tilde{f}}_{l}={\tilde{f}}_{l}\left(r+{\tilde{u}}_{l}^{k}\right).$$

Accordingly, the motion‐compensated reconstruction is given as

(4) $$f_{k,\text{MoCo}}=\sum_{l=1}^{K}{\tilde{T}}_{l}^{k}\circ {\tilde{f}}_{l}.$$

In order to achieve single‐view resolution, the proposed SAMoCo follows a similar strategy but drops the gating to avoid the downsides discussed in Section 1. Instead of applying transformations ${\tilde{T}}_{l}^{k}$ that map between gated reconstructions, we apply transformations $T_{i}^{n}$ to the (filtered) backprojection of single views ($X_{i}^{-1}p_{i}$), which we refer to as single‐angle reconstructions (SARs). Thus, the SAMoCo reconstruction is given as:

(5) $$f_{n,\text{SAMoCo}}=\sum_{i=1}^{N}T_{i}^{n}\circ X_{i}^{-1}p_{i}.$$

In that way any motion state of the scan can be reconstructed by setting the reference index n∈{1,⋯,N} accordingly.

#### 2.1.2 Practical realization – deep SAMoCo

**FIGURE 1:**
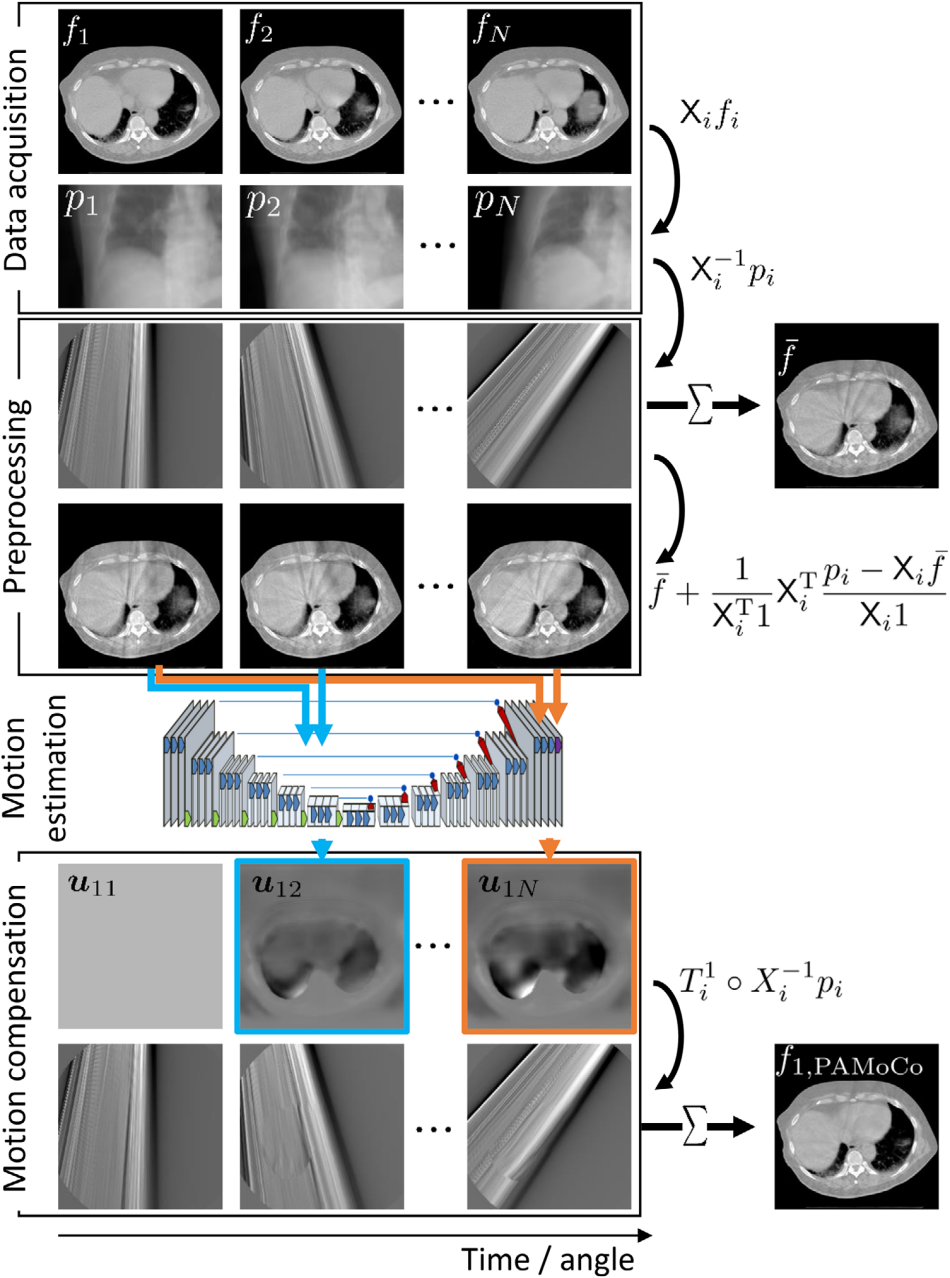
Workflow of the proposed Deep SAMoCo approach to reconstruct the 1st motion state. Any other motion state j can be reconstructed in the same way by changing the input to the network accordingly. Note that a shifted detector setup is considered. Therefore, the single angle reconstructions only cover about half the image. SAMoCo, single angle‐based motion compensation.

Implementing the motion compensation according to Equation (5), requires to know the transformations $T_{i}^{n}$ that map motion states $f_{i}$ to motion states $f_{n}$. Here, we aim to determine these transformations as a function of respective projections using a deep neural network as shown in Figure 1. In the Deep SAMoCo framework the network is trained to learn the following mapping:

(6) $$M_{\text{SAMoCo}}:\left[g_{i}\left(p_{i}\right),g_{n}\left(p_{n}\right)\right]\to u_{i}^{n},$$

Here, the function $g_{i}$ is designed to provide additional morphological information that is not present in $p_{i}$ or $p_{n}$. For that purpose, we chose $g_{i}$ to be the first update of an iterative reconstruction:

(7) $$g_{i}:p_{i}\to \bar{f}+\frac{1}{X_{i}^{T}1}X_{i}^{T}\frac{p_{i}-X_{i}\bar{f}}{X_{i}1},$$

where $\bar{f}$ is an initial (motion blurred) reconstruction that uses all available data, that is

(8) $$\bar{f}=\sum_{n=1}^{N}X_{n}^{-1}p_{n}.$$

In that way, the temporal information contained in $p_{i}$ is combined with the tomographic information of $\bar{f}$ to establish a robust mapping. Finally, the n ^th^ motion state $f_{n}$ is reconstructed by estimating N DVFs ${\left\{u_{i}^{n}\right\}}_{i\in \{1,\cdots ,N\}}$ and by applying them according to Equation (5).

Here, the deformation itself is realized via the spatial transformer function described in reference.^41^

#### 2.1.3 Deep SAMoCo network

Since it has shown good performance in other motion estimation tasks,^36^, ^37^, ^41^ a U‐Net‐like architecture is used to predict the DVF (required by Equation 5) that maps a given motion state $f_{i}$ to a reference motion state $f_{n}$.^42^ As $f_{i}$ and $f_{n}$ are to be estimated by the proposed approach, they are not available during inference time. Rather, modified SARs, $g_{i}\left(p_{i}\right)$ and $g_{n}\left(p_{n}\right)$, are provided as a two‐channel input to the network according to Equation (6).

The network itself consists of six stages with skip connections between the encoding and the decoding path. Each stage in the encoder and the decoder employs two convolutional layers (kernel size = 3 × 3 × 3) with a parametric rectified linear unit (PReLU) activation function.^43^ The number of filters of the convolutional layers is increased by a factor of two in each stage starting with 8 filters in the first one. To reduce the spatial dimensions, the encoding path uses 2 × 2 × 2 max pooling while the decoding path applies 2 × 2 × 2 nearest neighbor upsampling to get back to the initial dimensions. Note that the final convolution only uses three filters (and a linear activation) such that the three channels of the output represent the three spatial dimensions of the DVF.

#### 2.1.4 Data generation and training

To train the SAMoCo approach in a supervised setup, paired data $\left\{\left(g_{i}\left(p_{i}\right),g_{n}\left(p_{n}\right)\right),u_{i}^{n}\right\}$ consisting of the modified SAR inputs $\left(g_{i}\left(p_{i}\right),g_{n}\left(p_{n}\right)\right)$ and the corresponding ground truth vector field $u_{i}^{n}$ are required. Here, these data were generated using CBCT simulations based on a 4D clinical CT dataset. The dataset consists of respiratory‐gated CT scans (acquired with a Philips Brilliance Big Bore CT) of 84 patients who were examined for clinical indications and who in part had lung tumors.

Here each scan is reconstructed into 10 separate volumes, ${\hat{f}}_{i}$ with i∈{1,⋯,10}, representing 10 successive motion phases of the respiratory cycle. Due to the high temporal resolution of clinical CT, it can be assumed that these reconstructions are quasi‐static and free of motion artifacts. Therefore, they serve as prior volumes for the generation of paired training data.

Here, it has to be noted that we do not simulate CBCT scans which would include a continuous transition between these 10 reconstructions but just single samples of these motion states.

To do so, all reconstructions were resampled in a first step to a 320×256×160 grid with isotropic voxel size of 1.5 mm. In a second step, all possible DVFs $u_{i}^{n}$ between any two phases i,n∈{1,⋯,10} were calculated for the resampled reconstructions via deformable image registration. For the purpose of this study, this was performed using VoxelMorph.^41^ However, it has to be noted that the general concept does not rely particularly on VoxelMorph. Any other deformable image registration approach, for instance demons^44^ or DEEDS algorithm,^45^ could be used instead.

Finally, inputs to network $\left(g_{i}\left(p_{i}\right),g_{n}\left(p_{n}\right)\right)$ were generated by a forward projection in CBCT geometry, followed by a backprojection within the scope of the function $g_{i}$ as described by Equation (7). In any case, the projection operators were implemented to match the ones of the Varian TrueBeam system described in Section 2.3.3. With N views per CBCT scan (where N is typically in the order of several hundred) and 10 phases of the prior CT scans, there are 10N different realizations of $g_{i}\left(p_{i}\right)$ and thus (10${N)}^{2}$ different input combinations per patient. Since a precalculation of all realizations would lead to memory issues, they are rather calculated on‐the‐fly during training by randomly sampling two phases, i and j, as well two view angles, $\vartheta_{i}$ and $\vartheta_{n}$.

In that way 1000 random samples were drawn for each patient per epoch. Using 68 of the 84 patients (average lung volume: 3.54 ± 0.96 L, average difference between inspiration and expiration: 0.40 ± 0.12 L), the approach was trained for 500 epochs on four NVIDIA RTX 3090 GPUs using an Adam optimizer, a batch size of four, a learning rate of 0.0001, and the mean squared error between prediction and ground truth DVF as loss function. The network realization that performed best on another eight independent validation patients was used for performance evaluation on the remaining eight test patients (average lung volume: 3.39 ± 0.91 L, average difference between inspiration and expiration: 0.35 ± 0.08 L), as described in Section 2.3.2.

### 2.2 Residual artifact correction (RAC)

#### 2.2.1 Problem formulation

Let us assume we have an object with different motion states ${\left\{f_{i}\right\}}_{i\in \{1,\cdots ,N\}}$ and a set of transformations $T_{i}^{1}$ which map to the first motion state such that $f_{1}=T_{i}^{1}\circ f_{i}$. In that case, the SAR of $f_{1}$ is given as:

(9) $$X_{i}^{-1}X_{i}f_{1}=X_{i}^{-1}X_{i}\left(T_{i}^{1}\circ f_{i}\right).$$

where $X_{i}$ is the forward projection of the i ^th^ view and $X_{i}^{-1}$ the corresponding filtered backprojection (note that for a single view these operations do not cancel out). Summing up the SARs for all view angles yields:

(10) $$f_{1}=\sum_{i}X_{i}^{-1}X_{i}f_{1}=\sum_{i}X_{i}^{-1}X_{i}\left(T_{i}^{1}\circ f_{i}\right).$$

Within the deep SAMoCo framework, however, we do not have access to the $f_{i}$’s but only to their SARs $X_{i}^{-1}X_{i}f_{i}$. Therefore, we rather use the approximation $X_{i}^{-1}X_{i}\left(T_{i}^{1}\circ f_{i}\right)\approx T_{i}^{1}\circ \left(X_{i}^{-1}X_{i}f_{i}\right)$ and apply the transformation to the SARs instead. Thus, according to Equation (5), the deep SAMoCo for this example is given as

(11) $$f_{1,\text{SAMoCo}}=\sum_{i}T_{i}^{1}\circ \left(X_{i}^{-1}X_{i}f_{i}\right)\approx \sum_{i}X_{i}^{-1}X_{i}\left(T_{i}^{1}\circ f_{i}\right)=f_{1}.$$

Due to this approximation, some residual artifacts remain in our deep SAMoCo reconstruction even if the transformations are exactly know.

#### 2.2.2 Toy example

**FIGURE 2:**
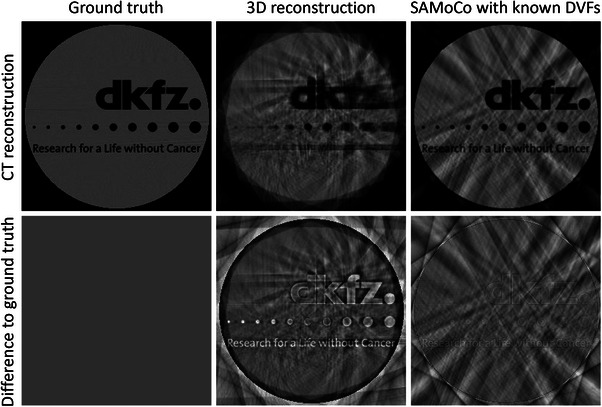
Toy example demonstrating the need for the proposed residual streak artifact correction. Motion‐corrupted projection data are generated by applying a view‐dependent axial scaling factor (1+0.1·sin(0.15·i)), with i denoting the view index, to the phantom prior to the forward projection. While a conventional 3D reconstruction shows severe motion artifacts (middle column), the SAMoCo using the known DVFs (right column) compensates for motion but contains residual streaks. DVF, displacement vector field; SAMoCo, single angle‐based motion compensation.

To illustrate the issue described in Section 2.2.1, Figure 2 provides the results of a toy example. Here, a cylindrical phantom containing the logo of our institution was scaled periodically to simulate motion‐corrupted projection data

(12) $$p_{i}=X_{i}T_{i}\circ f_{D},$$

with $f_{D}$ being the phantom and

(13) $$T_{i}:r\to \left(\begin{array}{ccc} 1+0.1\cdot \sin(0.15\cdot i) & 0 & 0\\ 0 & 1+0.1\cdot \sin(0.15\cdot i) & 0\\ 0 & 0 & 1 \end{array}\right)\cdot r,$$

representing the transformation that applies the periodic scaling in the axial plane. For our purpose, the motion frequency was chosen to correspond to a typical number of respiratory cycles during a 60 s CBCT scan. Due to the simplicity of $T_{i}$, the SAMoCo can be performed according to Equation (5) using the exact inverse of $T_{i}$. As shown in the right column of Figure 2, this yields an image similar to the ground truth but with residual streak artifacts.

#### 2.2.3 RAC network

To account for the residual streak artifacts A≡A(r), a second network is trained to learn the following mapping $M_{\text{RAC}}$:

(14) $$M_{\text{RAC}}:f_{n,\text{SAMoCo}}\to A,\;\;\text{s.t.}\;\;f_{n,\text{SAMoCo}}+A=f_{n}.$$

Here, the RAC network uses the same architecture as the SAMoCo network (see Section 2.1.3), except for the following two modifications: First, the RAC network uses a residual connection to estimate the artifacts A according to Equation (14), and second, it has only a single input channel ($f_{n,\text{SAMoCo}}$) and single output channel (A).

#### 2.2.4 Data generation and training

The generation of training data for the RAC network is based on the 4DCT dataset described in Section 2.1.4. For each training example, a random sequence $\left({\hat{f}}_{\nu_{1}},{\hat{f}}_{\nu_{2}},\cdots ,{\hat{f}}_{\nu_{N}}\right)$ was sampled, with $\nu_{i}\in \left\{1,\cdots ,10\right\}$ being a uniform random number and N=657 corresponding to the number of views. Subsequently, projection data were generated according to Equation (1) and reconstructed using the SAMoCo approach according to Equation (5). The corresponding artifact‐free labels were generated by performing an ideal motion compensation.

In total, 50 random sequences were simulated per patient. Here, 68 of the 84 patients were used for training, while 8 were used for validation and another 8 were reserved for testing. The network was trained for 300 epochs on an NVIDIA RTX 3090 GPU using an Adam optimizer, a batch size of one, a learning rate of 0.0001, and the mean squared error between the prediction end the ideal motion compensation as loss function.

### 2.3 Evaluation

#### 2.3.1 Accuracy of DVFs

The accuracy of the DVFs predicted by the deep SAMoCo is evaluated by a comparison against the ground truth DVFs (see Section 2.1.4) of the test set. To do so, ten random view angles were sampled for each patient and each phase of the prior 4DCT to generate projections $p_{i}$ according to Equation (1) and modified SARs $g_{i}$ according to Equation (7). Subsequently, the deep SAMoCo network was applied to predict DVFs according to Equation (6) between any combination of modified SARs.

**FIGURE 3:**
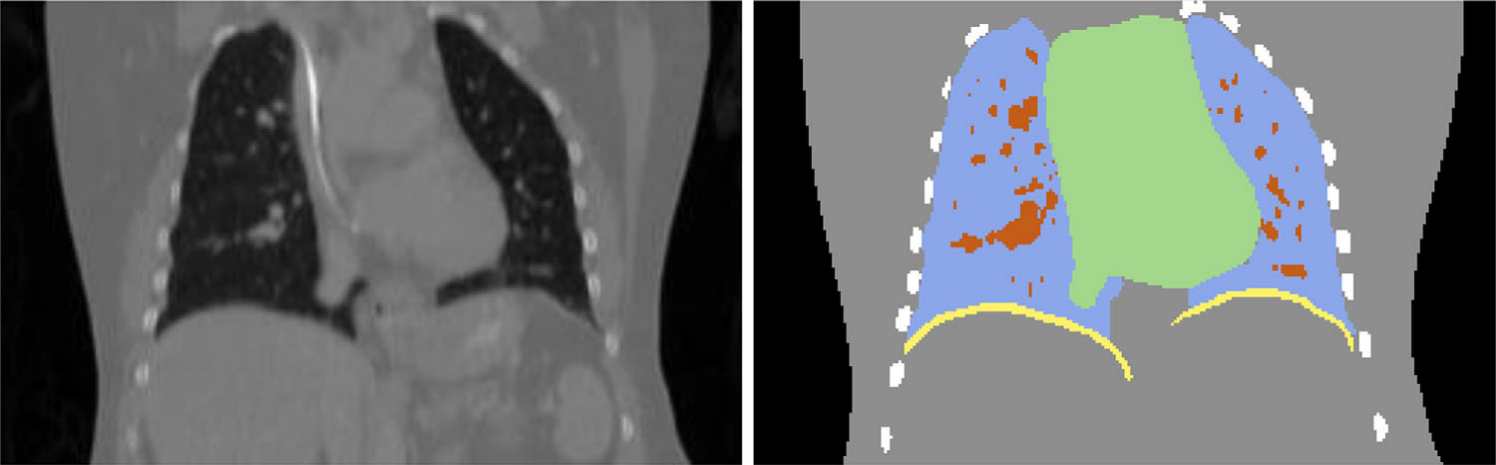
Segmentation to perform an anatomy‐specific evaluation of the DVFs. Blue: lung, orange: lung nodules, green: heart, white: ribs, yellow: diaphragm, gray: remainder. DVF, displacement vector field.

To further quantify the quality of the DVFs, an anatomy‐specific evaluation was performed. For that purpose the CT reconstructions were segmented using an internal segmentation tool. As shown in Figure 3, each voxel of the patient was assigned exclusively to one of the following classes: lung, lung nodules, heart, ribs, diaphragm, remainder.

Based on this segmentation, mean values of the predicted DVFs

(15) $${\bar{u}}_{c}=\frac{1}{K_{c}}\sum_{i=1}^{10}\sum_{n=1}^{10}\sum_{\nu_{i}=1}^{10}\sum_{\nu_{n}=1}^{10}\sum_{r}w_{n,c}\left(r\right)\cdot \left|u_{i}^{n}\left(r\right)\right|$$

as well as their mean absolute error with respect to the ground truth

(16) $$E_{c}=\frac{1}{K_{c}}\sum_{i=1}^{10}\sum_{n=1}^{10}\sum_{\nu_{i}=1}^{10}\sum_{\nu_{n}=1}^{10}\sum_{r}w_{n,c}\left(r\right)\cdot \left|u_{i}^{n}\left(r\right)-u_{i,\text{GT}}^{n}\left(r\right)\right|$$

were calculated, where $K_{c}=10^{4}\cdot \sum_{r}w_{n,c}\left(r\right)$ is a normalization constant, i and j are source and target motion phases, $\nu_{i}$ and $\nu_{j}$ refer to the corresponding random angles and $w_{n,c}\left(r\right)$ is the class‐ and motion state‐specific binary weight resulting from the segmentation.

#### 2.3.2 Simulation study

To test the proposed SAMoCo approach including the RAC network, simulations were performed using eight test patients of the 4DCT dataset described in Section 2.1.4. For each patient, CBCT projections were generated according to Equation (1) by a forward projection of a sequence of N (here: 657) prior volumes $\left(f_{1},f_{2},\cdots ,f_{N}\right)$ using the Varian TrueBeam geometry described in Section 2.3.3. Here, the n ^th^ prior volume $f_{n}$ is given as:

(17) $$f_{n}=\left(\left(\phi \left(n\right)-\Phi_{n}\right)\cdot T_{\Phi_{n}}^{\Phi_{n}+1}\right)\circ {\hat{f}}_{\Phi_{n}},$$

where ${\hat{f}}_{\Phi_{n}}$ is one of ten phases of the 4DCT dataset, ϕ(n):{1,⋯N}→[1,11) is a (non‐integer) phase signal representing the distribution of motion states during the scan, and $\Phi_{n}=\left\lfloor \phi \left(n\right)\right\rfloor$ is the greatest integer less than or equal to ϕ(n).

In that way we can mimic a continuous and smooth transition between different motion states as it would occur during a real CBCT scan.

**FIGURE 4:**
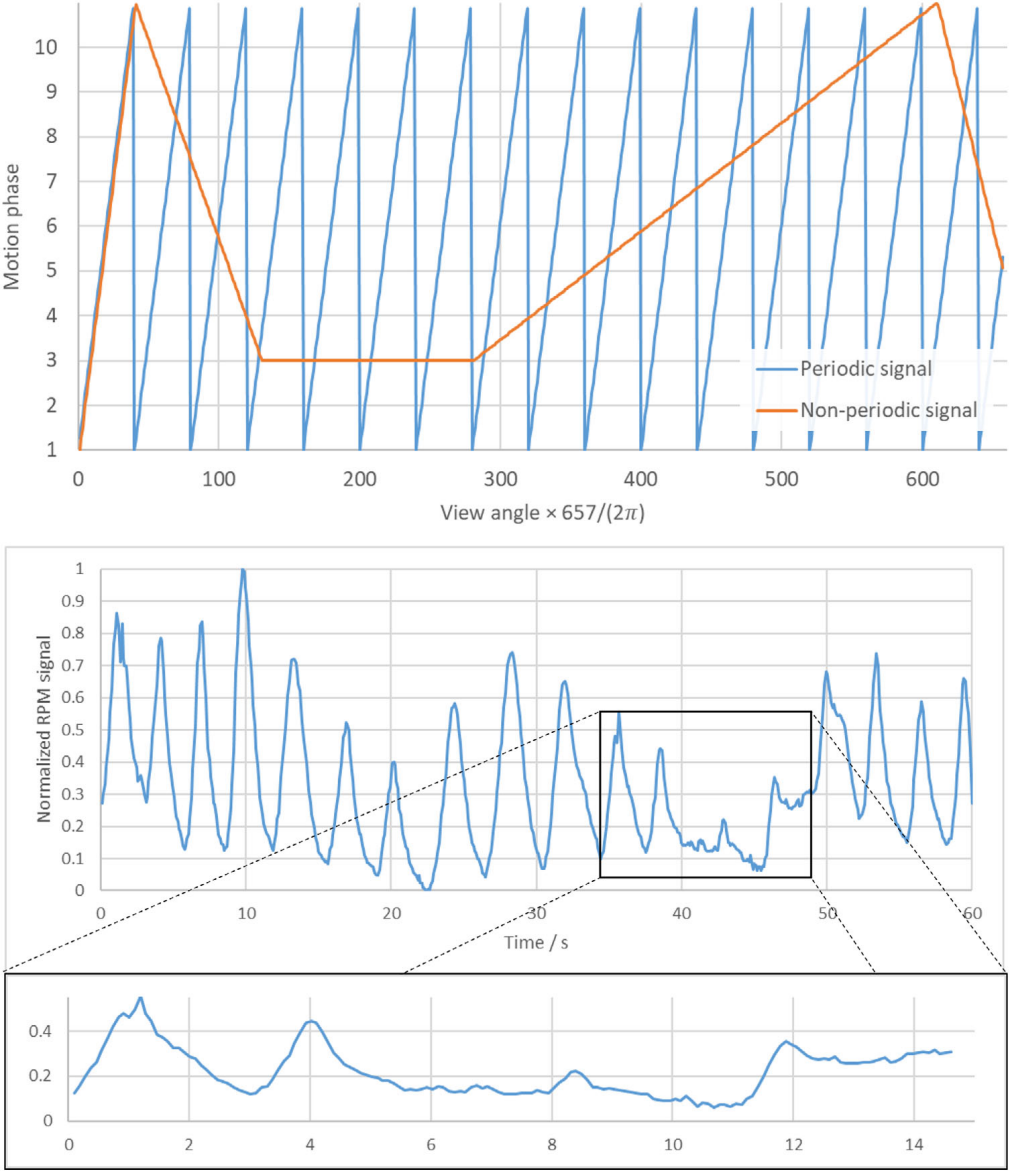
Top: Periodic and non‐periodic motion phases that were used for simulation. Bottom: Clinical example of a 60 s scan with a selected 15 s interval showing highly non‐periodic motion. Particularly in scans with such short acquisition times, the motion patterns may resemble the non‐periodic simulation case.

Following this strategy, a periodic and a non‐periodic motion pattern was simulated according to the phase signals shown in Figure 4. Here, the two scenarios can be thought as two extremal cases. While the periodic case corresponds to an ideal motion pattern with a respiration frequency similar to what is observed in most of our patient scans, the non‐periodic case shows a very complex motion pattern with a very limited number of respirations.

This can be seen as the result of a CBCT scan of a free‐breathing patient with a very short scan time as illustrated in Figure 4. Although this is currently a rather rare case, it may become more important in the future, especially with the trend towards systems with faster gantry rotation speed.^46^

Finally, the corresponding motion compensated reconstructions were evaluated in terms of their gray value accuracy as well as the accuracy of the position of the diaphragm. For the latter a sigmoid function

(18) $$s_{z_{0},h,c,b}\left(z\right)=\frac{h}{1+\exp(-c\cdot \left(z-z_{0}\right))}+b,$$

with open parameters z0,h,c, and b was fitted to line profiles (extracted from the motion compensated reconstructions) that run perpendicular to the surface of the diaphragm. In that way, the fitted inflection point $z_{0}$ can be used as a measure of the diaphragm position.

Here, 400 line profiles were evaluated and averaged for each patient and each motion compensated reconstruction $f_{n,\text{SAMoCo}}$. For comparison the same evaluation was performed for the ground truth, that is, $f_{n}$ as given in Equation (17).

#### 2.3.3 Pilot patient study

Patient measurements were performed using the kV imaging unit of a Varian TrueBeam system. Here, the source to detector distance is 1500 mm and the source to isocenter distance is 1000 mm. All scans were performed at 125 kV with the 2 × 2 binning mode in which the CsI‐based detector (PaxScan 4030) has 1024 × 768 pixels with an effective pixel size of 0.388 mm×0.388 mm. To increase the field of measurement to about 46 cm, the system was operated in shifted‐detector mode in which the detector is laterally off‐centered by 160 mm. In total, 660 to 840 projections were acquired over 360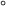 with a frame rate between 7  and 11 fps, leading to a scan time of 60 s to 120 s. To monitor respiratory motion, the Varian Real‐time Position Management (RPM) systems was synchronized with the CBCT scan.

**FIGURE 5:**
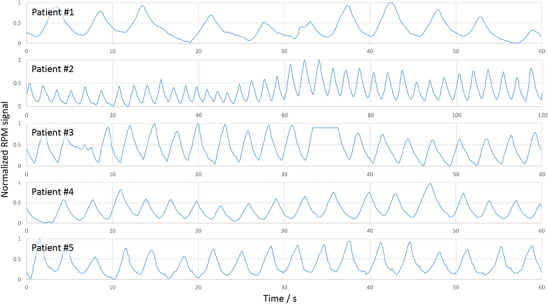
RPM signals of the five patient measurements evaluated in this study. Exemplary reconstructions for the first two patients can be found in Figure 9. RPM, real‐time position management.

For this study measurements of five patients were evaluated. Since there is no ground truth, the position of the diaphragm was determined as a function of the scan time (see Section 2.3.2) and compared quantitatively to the signals (see Figure 5) of the RPM system.

## 3 RESULTS

### 3.1 Accuracy of DVFs

**FIGURE 6:**
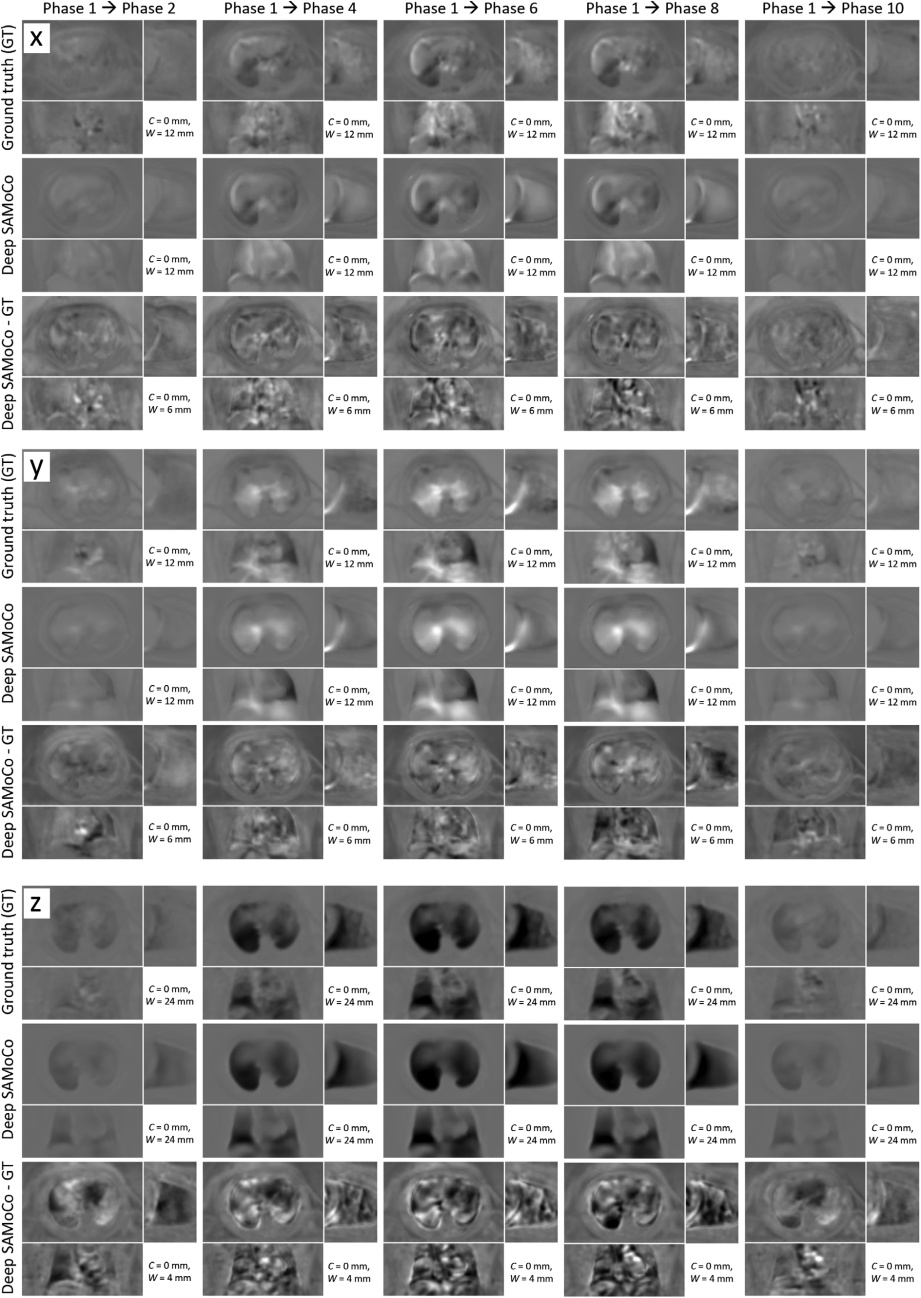
x‐, y‐, and z‐component (top, middle, bottom) of the ground truth DVFs, the corresponding deep SAMoCo prediction, as well as their difference. It has to be noted that this figure shows only a subset of the predicted DVFs (phase 1 → 2, 1 → 4, 1 → 6, 1 → 8, 1 → 10), while DVFs were calculated between any combination of the 10 phases of the 4DCT prior. DVF, displacement vector field; SAMoCo, single angle‐based motion compensation.

The accuracy of the predicted DVFs was evaluated as described in Section 2.3.1. As shown for one exemplary test patient in Figure 6, the predicted DVFs of the deep SAMoCo are in good agreement with the ground truth. However, since DVFs are predicted as a function of only two projections, high frequencies of the DVFs are reproduced with reduced accuracy. The quantitative analysis summarized in Table 1 shows similar trends. While the anatomy‐specific mean values of the predicted DVFs differ by less than 10% from the corresponding ground truth, the evaluation of the mean absolute error on a pixel level yields slightly higher deviations. Nevertheless, these deviations are on average well below the voxel size for all anatomies. Here, the highest errors occur in the heart, which is most likely due to the superposition of respiratory and cardiac motion. Since our training strategy is focused on the respiratory motion, it is not optimal for estimating cardiac motion and leads to a reduced accuracy within the heart.

**TABLE 1:** Anatomy‐specific mean of the DVFs of the GT and the deep SAMoCo as well as the (pixelwise) MAE for the eight patients of the simulated test data set according to Equations (15) and (16).

	Mean GT/mm	Mean deep SAMoCo/mm	MAE/mm
Lung	1.50 ± 1.04	1.49 ± 1.11	0.73 ± 0.23
Lung nodules	1.81 ± 1.39	1.87 ± 1.49	0.79 ± 0.28
Heart	1.72 ± 1.00	1.56 ± 1.09	1.06 ± 0.30
Ribs	0.45 ± 0.27	0.41 ± 0.22	0.32 ± 0.09
Diaphragm	2.69 ± 2.08	2.80 ± 2.23	0.86 ± 0.33
Remainder	0.94 ± 0.49	0.84 ± 0.48	0.53 ± 0.12

Abbreviations: DVF, displacement vector field; GT, ground truth; MAE, mean absolute error; SAMoCo, single angle‐based motion compensation.

### 3.2 Simulation study

**FIGURE 7:**
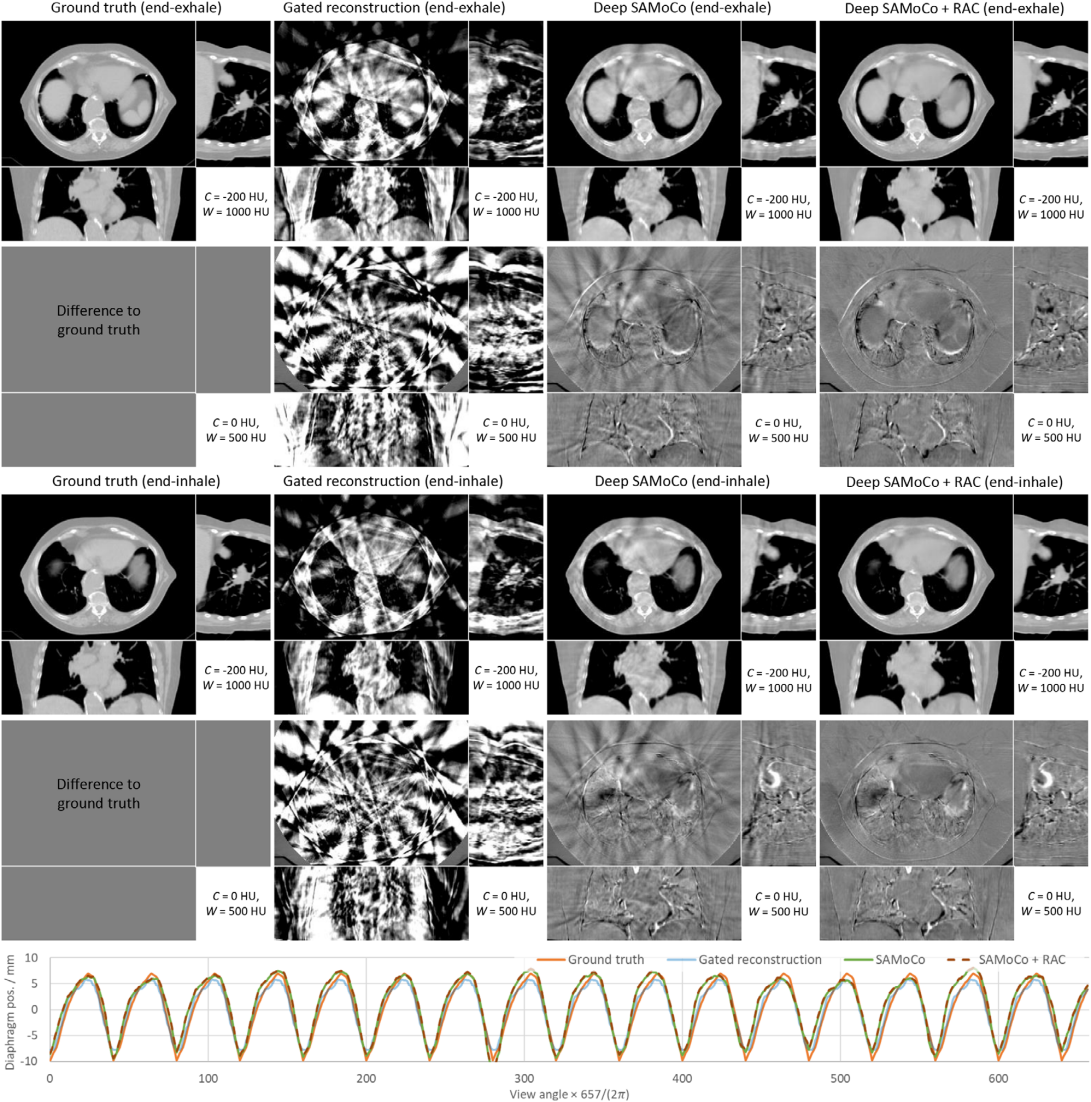
Simulation study for a periodic motion signal. The CT images show motion compensated reconstructions for two motion states (top: end‐exhale, middle: end‐inhale). The diaphragm position according to Section 2.3.2 is shown below for all views or motion states respectively.

Motion‐compensated reconstructions were generated as described in Section 2.1 using the data introduced in Section 2.3.2. In addition, gated reconstructions were performed according to Equation (2) as a reference. Here, the retrospective  gating is based on the phase signals shown in Figure 4, that is, for every time point all data within a 20% window centered around the respective phase were used for reconstruction. The corresponding results for a periodic test case are shown in Figure 7. Since the retrospective  gating leads to sparse angular sampling, the corresponding gated reconstructions suffer from severe sparse‐view artifacts. However, despite the poor image quality, high contrast structures such as the diaphragm are clearly resolved. Therefore, its position can still be determined quite accurately (see Figure 7, bottom curve).

The Deep SAMoCo, in contrast, is designed to use all data by estimating and applying DVFs that account for the present motion. As a results, the corresponding reconstructions show highly improved image quality. Even though no gating information is used within the SAMoCo framework, all present motion states or the diaphragm positions can be resolved with high accuracy. However, due to inconsistencies discussed in Section 2.2, some streak artifacts still remain. Therefore, a further improvement can be achieved by applying RAC network which is trained to address this issue. In fact, the SAMoCo + RAC reconstructions are almost free of artifacts and only show some blurring in the region of the heart.

**FIGURE 8:**
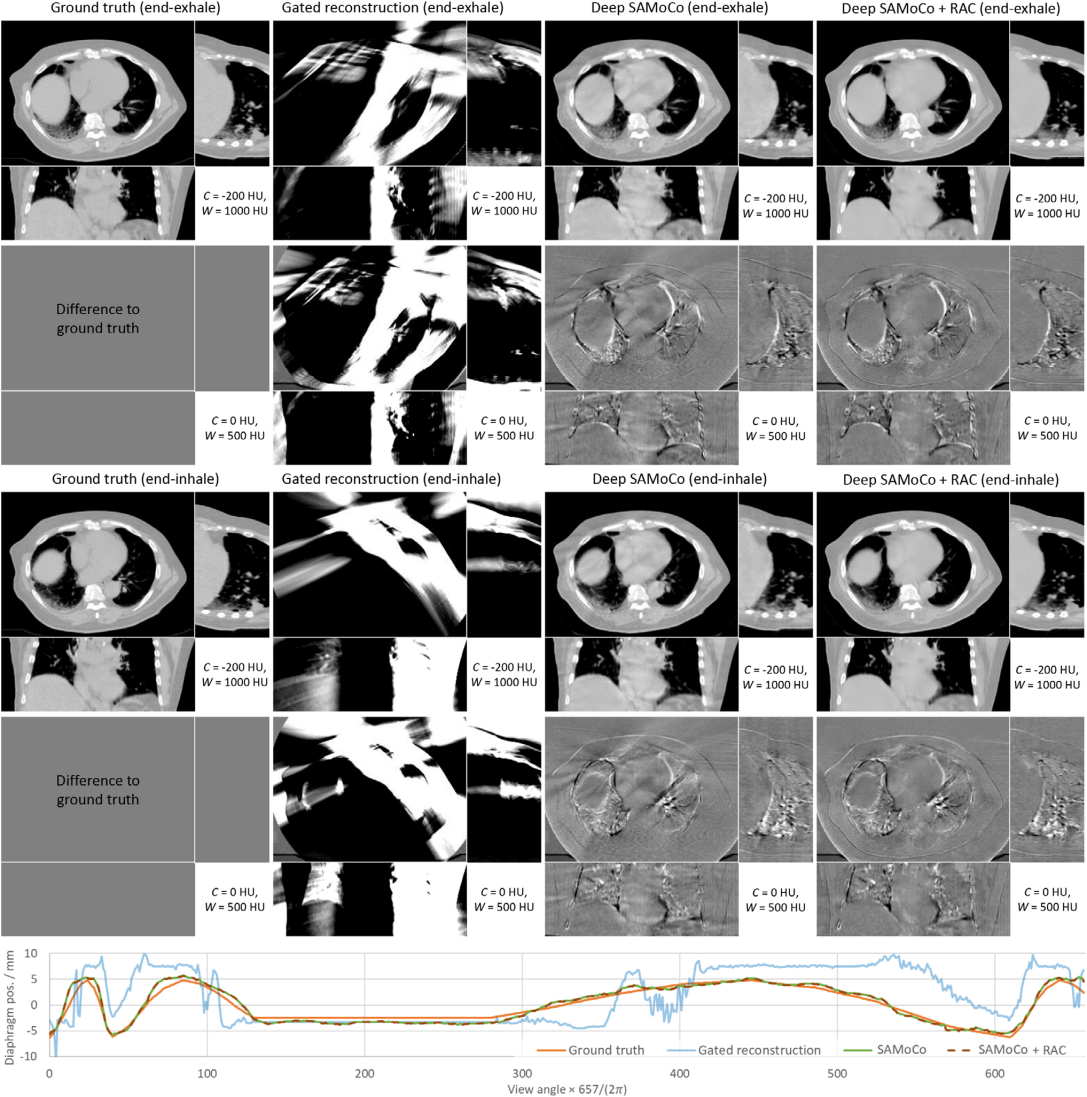
Simulation study for a non‐periodic motion signal. The CT images show motion compensated reconstructions for two motion states (top: end‐exhale, middle: end‐inhale). The diaphragm position according to Section 2.3.2 is shown below for all views or motion states respectively.

Similar results can be obtained for the non‐periodic case shown in Figure 8. As the Deep SAMoCo is trained to estimate DVFs between arbitrary motion states independent of their temporal distribution, it can handle periodic and non‐periodic cases in the same way and with the same accuracy. This becomes evident when considering the evaluation of the diaphragm position, as shown in the bottom curve. Similar to the periodic case, the application of the RAC network can further boost image quality without affecting the accuracy of the motion compensation. Comparing these results with those of the gated reconstruction, the advantages of the proposed approach become particularly clear. Since any gating strategy relies on different views sharing the same motion state, it fails in case of non‐periodic motion patterns. This downside is reflected by the poor image quality that does not allow a reasonable determination of the diaphragm position.

A more quantitative evaluation is given in Table 2. Considering the average deviation of the diaphragm position as well as the mean absolute error of the CT values, there is a good agreement with the quantitative findings discussed above. While the gated reconstruction yields poor image quality, it is yet able to provide accurate diaphragm positions for the periodic case. As expected, it fails completely in the non‐periodic case. The SAMoCo, in contrast, provides a similar accuracy for both cases. As intended, the RAC network does not affect the accuracy of the diaphragm position but leads to an improved image quality.

**TABLE 2:** Deviation from the ground truth averaged over the eight patients of the simulated test data set.

	Diaphragm position/mm		Mean absolute error/HU	
	periodic	non‐periodic	periodic	non‐periodic
Gated reco.	**0.49** ± **0.17**	3.79 ± 1.32	245 ± 21	1120 ± 87
SAMoCo	0.73 ± 0.24	**0.74** ± **0.31**	35 ± 5	33 ± 5
SAMoCo+RAC	0.73 ± 0.25	0.75 ± 0.31	**27** ± **5**	**26** ± **4**

Abbreviations: RAC, residual artifact correction; SAMoCo, single angle‐based motion compensation.

### 3.3 Pilot patient study

**FIGURE 9:**
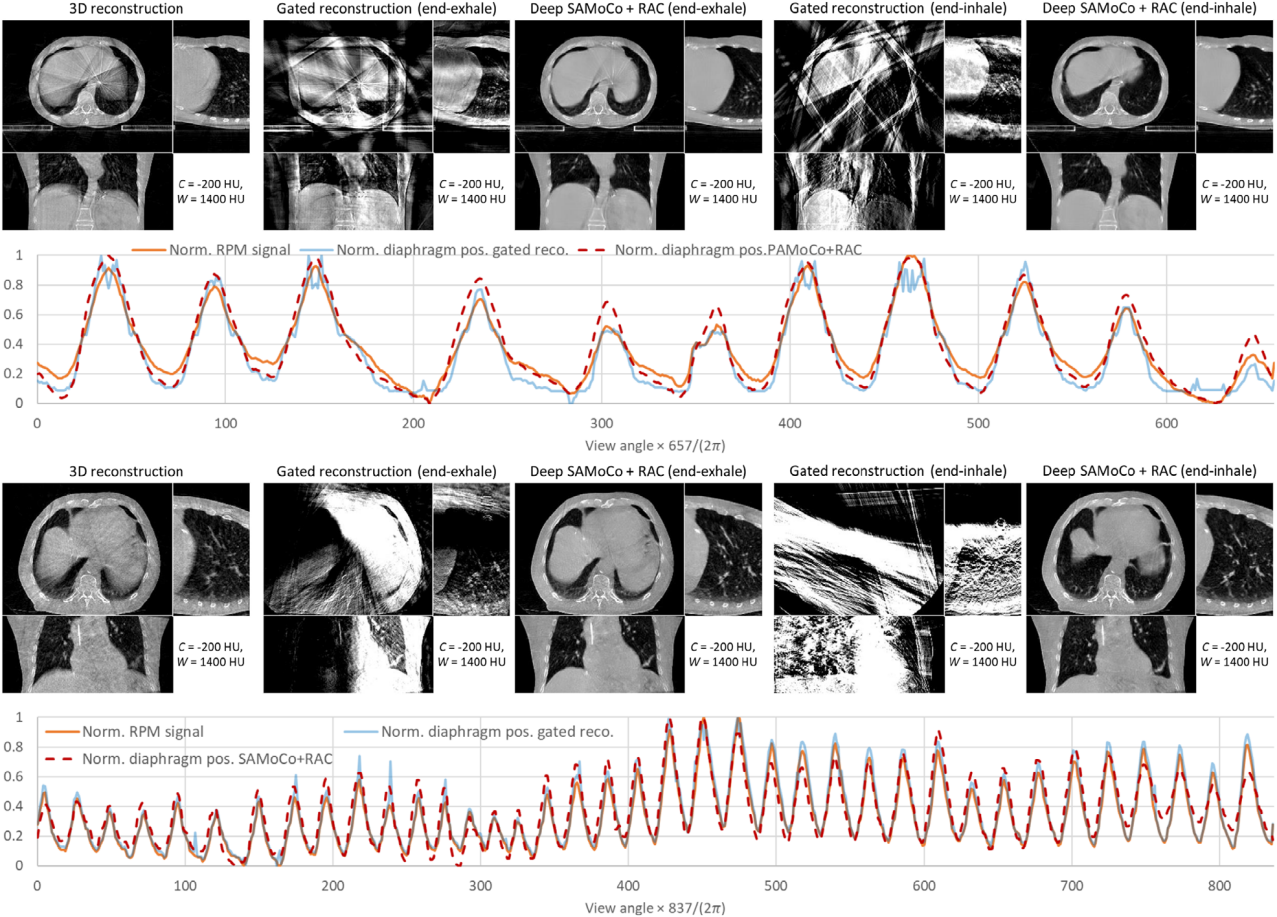
Results for a 1 min CBCT scan (top) and a two minute CBCT scan (bottom) of two different patients. The CT images show the 3D CBCT reconstruction that is corrupted by motion as well as motion compensated reconstructions for two motion states (left: end‐exhale, right: end‐inhale). As there is no ground truth, the normalized RPM signal is plotted against the normalized diaphragm position (according to Section 2.3.2) below.

Patient measurements were performed according to Section 2.3.3. Similar to the simulation study, gated reconstructions were used as reference. Here, however, the retrospective  gating was performed according to the external RPM signal. The corresponding reconstructions as well as the results of the SAMoCo including the RAC network are shown in Figure 9 for two different patients. Similar to the previous experiments, the gated reconstructions suffer from a poor image quality. Especially in the extremal motion states (end‐exhale and end‐inhale), there are very few projections that can be used if the respiration is not perfectly regular. In particular this can be observed in the second case in which the motion pattern changes strongly during the scan. The Deep SAMoCo, in contrast, is not affected by the motion pattern and yields constant image quality independent of the motion state or the view angle respectively.

To further assess the quality of the SAMoCo reconstructions, the diaphragm position was evaluated as a function of the scan time or the view angle respectively and compared against the RPM signal. Even though the RPM signal actually represents the motion of a marker block on the patient's chest, we expect a high correlation once both signals are normalized to be in the same range (here: [0,1]). The corresponding comparison is plotted below the CT images in Figure 9 and indeed shows a good agreement with the RMP signal as well as the curve of the gated reconstruction.

Comparing the normalized diaphragm positions to the normalized RPM signal of all test patients yields an average deviation of 7.5% ± 1.4% for the gated reconstruction and 7.1% ± 1.2% for the SAMoCo as well as the SAMoCo with RAC.

## 4 DISCUSSION AND CONCLUSION

Current 4D motion compensation approaches usually rely on some kind of gating strategy. However, especially in case of irregular or non‐periodic motion patterns, these strategies have major downsides as demonstrated by our experiments. Even though the gated reconstructions shown here are the simplest representatives of this class of approaches, we expect a similar behavior for more sophisticated gating‐based motion compensation strategies. Since they are typically designed to operate on a set of gated reconstructions (either by estimating DVFs as, for example, in refs. [30, 31] or by applying some sort of post‐processing as, for example, in refs. [22, 23, 24]), the gated reconstructions must have a certain image quality in the first place. If this is the case, advanced approaches as discussed in Section 1 are most likely to provide as high image quality as the proposed deep SAMoCo. However, if only very few projections share the same motion state, this criterion can certainly not be met.

To address this issue, the deep SAMoCo approach does not rely on gating at all, but is trained to estimate DVFs on a single‐view level as a function of so‐called SARs. In practice, this offers several advantages. First of all, not requiring gating signals simplifies the clinical workflow and reduces the time needed for patient preparation. Second, operating on a single‐view level provides a temporal resolution that corresponds to the acquisition time of a single projection image. Last and most importantly, the ability to estimate DVFs from any two SARs regardless of their motion states makes the deep SAMoCo applicable to arbitrary motion patterns. Therefore, the deep SAMoCo is particularly useful for cases with unsteady breathing, compensation of residual motion during a breath‐hold scan, or scans with fast gantry rotation times in which the data acquisition only covers a very limited number of breathing cycles. Furthermore, this allows to update an existing patient model, for example, a planning CT or a previously acquired CBCT, to the current motion state by only acquiring one additional x‐ray image. Thus, such an update can be performed almost in real time with minimal effort and minimal dose, offering new possibilities for image‐guided radiation therapy or interventional procedures. Nevertheless, it has to be noted that it works at least just as well for periodic signals as existing gating‐based methods.

These advantages are confirmed by our experiments.

Evaluating the quality of the predicted DVFs shows a high similarity to the ground truth with average errors well below the voxel size used in our experiments. In particular, the DVF estimates show a high accuracy for different anatomical regions with slightly higher deviations in the heart due to the superposition of respiratory and cardiac motion. Applied to periodic and non‐periodic CBCT simulations, the deep SAMoCo shows an equally well performance and provides motion‐compensated reconstructions that deviate from the ground truth by less than 35 HU on average without the RAC network and less than 27 HU with the RAC network. In both cases, these reconstructions allowed an accurate determination of the diaphragm position, which was used as an additional performance measure. Independent of the motion pattern, it could be determined with an accuracy of about 0.75 mm, which corresponds to half the voxel size.

Similar results could be obtained for real measurements at a Varian TrueBeam system. Even for patients with a very irregular breathing, the deep SAMoCo provides constant image quality across all motion states and a high correlation of the extracted diaphragm positions to the external RPM signal.

Finally, it has to be noted that even though the focus of this study is on respiratory motion compensation, the general concept of the deep SAMoCo applies similarly to other types of motion. If appropriate training data are available, it may be used, for example, to compensate for cardiac motion in CT and CBCT, to handle motion in interventional radiology, or to compensate for head motion in dental CBCT.

## CONFLICT OF INTEREST STATEMENT

The authors declare no conflicts of interest.
